# Primary malignant tumors of the adrenal glands

**DOI:** 10.6061/clinics/2018/e756s

**Published:** 2018-11-27

**Authors:** Madson Q Almeida, Joao Evangelista Bezerra-Neto, Berenice B Mendonça, Ana Claudia Latronico, Maria Candida B V Fragoso

**Affiliations:** I Unidade de Suprarrenal, Laboratorio de Hormonios e Genetica Molecular LIM/42, Servico de Endocrinologia e Metabologia, Hospital das Clinicas HCFMUSP, Faculdade de Medicina, Universidade de Sao Paulo, Sao Paulo, SP, BR; II Instituto do Cancer do Estado de Sao Paulo (ICESP), Hospital das Clinicas HCFMUSP, Faculdade de Medicina, Universidade de Sao Paulo, Sao Paulo, SP, BR

**Keywords:** Adrenocortical Carcinoma, Pheochromocytoma, Paraganglioma, Treatment

## Abstract

Malignancy must be considered in the management of adrenal lesions, including those incidentally identified on imaging studies. Adrenocortical carcinomas (ACCs) are rare tumors with an estimated annual incidence of 0.7–2 cases per year and a worldwide prevalence of 4–12 cases per million/year. However, a much higher incidence of these tumors (>15 times) has been demonstrated in south and southeastern Brazil. Most ACCs cause hypersecretion of steroids including glucocorticoids and androgens. ACC patients have a very poor prognosis with a 5-year overall survival (OS) below 30% in most series. Pheochromocytoma or paraganglioma (PPGL) is a metabolically active tumor originating from the chromaffin cells of the adrenal medulla. The incidence of PPGL is 0.2 to 0.9 cases per 100,000 individuals per year. Pheochromocytomas are present in approximately 4-7% of patients with adrenal incidentalomas. Classically, PPGL manifests as paroxysmal attacks of the following 4 symptoms: headaches, diaphoresis, palpitations, and severe hypertensive episodes. The diagnosis of malignant PPGL relies on the presence of local invasion or metastasis. In this review, we present the clinical and biochemical characteristics and pathogenesis of malignant primary lesions that affect the cortex and medulla of human adrenal glands.

## INTRODUCTION

Human adrenal glands can be affected by several malignant conditions. Indeed, malignancy must be considered in the management of adrenal lesions, including those incidentally identified on imaging studies (adrenal incidentalomas). Adrenal incidentalomas are identified in 1–4% of abdominal imaging studies, and the incidence of adrenal incidentaloma increases with age. In medical series, most adrenal incidentalomas are adenomas (80%), and only 8% are carcinomas. Therefore, the incidental discovery of an adrenal mass could be considered an opportunity to offer a curative approach for malignant lesions 1.

Adrenocortical carcinomas (ACCs) are rare tumors with an estimated annual incidence of 0.7–2 cases per year and a worldwide prevalence of 4–12 cases per million/year 2. However, a much higher incidence of adrenal tumors (>15 times) has been demonstrated in the pediatric population in south and southeastern Brazil, and these tumors are associated with a specific germline mutation in *TP53* 3,4. These malignant tumors can be diagnosed by endocrine signs of steroid excess, compression symptoms due to tumor growth or during evaluation of adrenal incidentalomas. The majority of adrenal tumors secrete excess cortisol, although this cortisol hypersecretion can be subclinical. ACC patients have a very poor prognosis with a 5-year overall survival (OS) below 30% in most series. Despite this unfavorable outcome, some patients are cured after surgical removal, and others have relatively slow metastatic progression. The recent discovery of genetic alterations underlying the pathogenesis of ACC have led to the identification of ACC subgroups with dismal prognoses 5.

Pheochromocytoma or paraganglioma (PPGL) is a metabolically active tumor originating from the chromaffin cells of the adrenal medulla. The incidence of PPGL is 0.2 to 0.9 cases per 100,000 individuals per year. Pheochromocytomas are present in approximately 4-7% of patients with adrenal incidentalomas. Classically, PPGL manifests as paroxysmal attacks of the following 4 symptoms: headaches, diaphoresis, palpitations, and severe hypertensive episodes. The diagnosis of malignant PPGL relies on the presence of local invasion or metastasis. The most common sites of metastasis include bones, lymph nodes and the liver. In this review, we present the clinical and biochemical characteristics and pathogenesis of malignant primary lesions that affect the cortex and medulla of human adrenal glands 6.

### Adrenocortical carcinoma

ACC presents a bimodal distribution, with the first peak in children <5 years and the second 7 peak during the 4^th^ and 5^th^ decades of life and a slight predominance in women 2,8. In the pediatric population, only 25 new cases are diagnosed each year in the USA. In south and southeastern Brazil, approximately 78% of children and 13% of adults with adrenal tumors harbor a specific germline mutation (p.R337H) of the p53 tumor suppressor 9,10. A founder effect has been demonstrated in the great majority of pediatric Brazilian patients with adrenocortical tumors caused by this p53 mutation 11.

Virilization syndrome is the most frequent presentation of adrenal tumors during childhood. Hypercortisolism can be associated with androgen overproduction, mainly of dehydroepiandrosterone sulfate (DHEAS) and testosterone, leading to gonadotropin-independent precocious puberty 3,12. In adults, cortisol hypersecretion is the most common hormone disorder. Adult ACC can also be associated with virilization alone or a mixed syndrome (virilization and Cushing's syndrome). Estrogen and aldosterone overproduction are highly uncommon 2. Although uncommon, patients with advanced disease can present with cachexia and body weight loss 13.

Most ACCs are sporadic, but some inherited disorders are associated with a high incidence of ACC, such as Li-Fraumeni syndrome (LFS), Beckwith-Wiedemann syndrome (BWS), Lynch syndrome (LS) and multiple endocrine neoplasia syndrome type 1 (MEN1) 14 (Table 1). LFS is a cancer predisposition syndrome usually caused by an inherited *TP53* gene mutation and is characterized by a high occurrence of sarcomas, early onset breast cancer, brain cancer and leukemia 15. ACC accounts for approximately 3% to 10% of LFS-associated cancers 5.

Somatic *TP53* mutations have been identified in 25–35% of sporadic ACCs. Nevertheless, these mutations represent a late event during carcinogenesis and have been associated with larger tumors and more advanced disease 14. Somatic mutations in the *TP53* gene are a predictor of poor overall survival in adults with ACC 16.

The overexpression of *IGF2* is one of the most important mechanisms of adrenocortical tumorigenesis. IGF2 is a potent *in vitro* growth promoter of adrenal cortex carcinoma cell lines 17. The *IGF2* gene is located on chromosome 11p15 and encodes a growth factor that is expressed only through the inherited paternal allele, while the maternal allele is silenced. Genetic or epigenetic alterations in the 11p15 region may alter the expression of the *CDKN1C, IGF2* and *H19* genes, which are structurally organized in a cluster, increasing *IGF2* expression and inactivating the *CDKN1C* and *H19* genes, which are a negative cell cycle regulator and a transcriptional repressor of *IGF2*, respectively. BWS is an example of epigenetic alterations in the 11p15 region 18. Macrosomia, macroglossia, visceromegaly, neonatal hypoglycemia, and ear and abdominal wall abnormalities characterize BWS. Children with BWS also have a high risk of developing Wilms' tumors, hepatoblastomas, rhabdomyosarcomas, and ACCs 19.

The gene expression profiles of 94 ACCs in adults demonstrated that the *DGL7* and *PINK1* genes were differentially expressed between recurrent and nonrecurrent tumors 20. Furthermore, the difference between *BUB1B* and *PINK1* gene expression was a significant predictor of OS in adults. The prognostic importance of the combined expression of these genes was subsequently validated in adult ACC patients from our institution but not in pediatric patients 21.

### Diagnostic evaluation

Autonomous cortisol secretion may be identified in almost 70% of patients with ACC, although this finding is clinically unapparent in many patients 22. The European Network for the Study of Adrenal Tumors (e-mail: ENS@T) has suggested a comprehensive hormone investigation for cortisol oversecretion (1 mg dexamethasone supression test at 23 h), basal cortisol and ACTH levels, and free 24-h urinary cortisol concentrations), excess of sexual steroid and steroid precursors [DHEA-S, 17-OH-progesterone, androstenedione, testosterone and 17-β-estradiol levels (only in men and postmenopausal women)], primary aldosteronism (aldosterone and renin levels in patients with hypertension and/or hypokalemia) and pheochromocytoma (total 24-h urinary metanephrine or plasma free metanephrine concentrations) 23. In our center, 11-deoxycortisol levels are also evaluated in patients with masses suspicious for malignancy due to the prognostic value of this parameter.

Most ACCs are large, heterogeneous lesions with irregular contrast uptake (Table 2). On CT, ACC is characterized by a precontrast density greater than 10 Hounsfield units (HU), indicating low lipid content, and absolute contrast washout <40% to 50% 1. In fact, malignant primary adrenal lesions usually have a precontrast density >30 HU. Magnetic resonance imaging (MRI) with gadolinium has the same accuracy as CT but may be superior for evaluating vascular invasions. Positron emission tomography (PET) using 18F-fluorodeoxyglucose (FDG) coupled to a CT scan is helpful for evaluating adrenal tumors. Malignant lesions have high metabolic activity leading to high FDG uptake. Adrenal adenomas usually have low FDG uptake. However, pheochromocytomas, adrenal metastases and rarely adenomas (especially those with high hormonal activity) can have high metabolic activity, resulting in false positives 1.

A careful histopathological evaluation is critical for the correct diagnosis of adrenal tumors, especially for neoplasms localized to the adrenal gland 24. The first step of the analysis consists of evaluating several macroscopic parameters, including tumor size and weight, capsule integrity, and the presence of hemorrhage or necrosis. The second step is to analyze several microscopic aspects that, together, confirm the diagnosis 25. The Weiss score comprises nine histopathological parameters: three are related to tumor structure (presence of necrosis, diffuse architecture and percentage of clear cells), three are related to cellular structure (atypical mitoses, number of mitoses and high nuclear grade (Fuhrman 3-4)), and three are related to invasion (vascular invasion, sinusoidal invasion and capsular invasion). According to the Weiss system, a tumor of the adrenal cortex is defined as carcinoma when it presents with at least three of these criteria 26.

Ki67 is a protein expressed in all cell cycle phases except G0 and represents a validated cell proliferation index. Ki67 is an important prognostic factor for localized ACCs. When evaluated by immunohistochemistry, a Ki67 index >10% can differentiate tumors with a high risk of recurrence that are potentially candidates for adjuvant therapy. High levels of Ki67 have also been associated with worse OS in patients with advanced disease and can help in decision-making regarding treatment 27.

### Staging

The staging system proposed by the e-mail: ENS@T is the best available system, as this system clearly identifies patient prognosis 22. The e-mail: ENS@T system classifies tumors as stage I or II when they are restricted to the gland and <5 cm or >5 cm in size, respectively. Stage III is determined by adjacent tissue invasion, neoplastic thrombus to the vena cava or renal vein, or regional lymph node metastasis. In addition, stage IV is determined by the presence of distant metastasis.

In 2015, the e-mail: ENS@T proposed that metastatic ACC should be classified into three subgroups (IVa, IVb and IVc). Subgroup IVa included patients with regional lymph node involvement and patients with one or 2 metastatic organs, including lymph nodes. Subgroups IVb and IVc included patients with 3 or more metastatic organs, respectively. Furthermore, some factors associated with worse prognoses are represented in the GRAS score (Grade: Weiss >6 and/or Ki67 >20%; R status: uncomplete resection of the primary tumor; **A**ge: age greater than or equal to 50 years; and **S**ymptoms: symptoms related to the tumor or hormonal production). The combination of the e-mail: ENS@T classification system and the GRAS score have a good correlation with patient prognosis in stage III and IV adrenal carcinomas 28.

Staging is the most important prognostic factor for ACC patients. There is still considerable variability among published studies about the likelihood of recurrence and survival. In recent studies, patients with stage I disease had an estimated 5-year OS rate of 66% to 82%. In patients with stage II and III disease, the 5-year OS rate varied from 58% to 64% and 24% to 50%, respectively. In patients with stage IV disease, the estimate 5-year OS rate decreased significantly to 0% to 17%. Other predictors of poor prognosis are older age at diagnosis, mixed syndrome (cortisol and androgen production) or estrogen-producing ACC, and incomplete resection, either with gross (R2) or microscopic residual disease (R1) 28.

### Treatment

Complete surgical resection is the most effective treatment 2. Unfortunately, complete resection is achievable only for locally restricted disease (stages I, II and some stage III tumors) 29. Biopsy of adrenal masses is indicated to confirm the anatomopathological diagnosis of noncortical adrenal lesions or if surgery is not the initial approach, but the anatomopathological diagnosis is necessary before initiating systemic therapy for advanced metastatic disease.

Experienced surgeons should perform surgery with curative intent to avoid incomplete resections and/or rupture of the tumor capsule. A laparoscopic approach is indicated for tumors <6 cm in size without signs of local invasion and should only be performed in highly experienced centers. *En bloc* resection is recommended when there is adjacent tissue invasion 29. The presence of a vena cava thrombus is compatible with complete resection, but extracorporeal circulation might be necessary. Prophylactic lymphadenectomy is not clearly associated with better prognosis and is not mandatory 29.

Patients with a suspected adrenal mass and abnormal glucocorticoid secretion, whether clinical or subclinical, should receive exogenous glucocorticoid replacement during the perioperative and postoperative periods to prevent acute adrenal insufficiency after resection of the tumor. We recommend that 100 mg of intravenous (*i.v*) hydrocortisone be administered at the time of anesthetic induction followed by 50 mg of *i.v* hydrocortisone every 8 h for approximately 2 days, after which hydrocortisone replacement can be provided by oral therapy.

After a complete surgical resection, patients should be followed up every 3 months. The follow-up of these patients should include a complete physical examination, hormonal evaluation and complete radiological evaluation including CT of the thorax and abdomen. After 2-3 years of follow-up without recurrence, imaging studies can be performed every 6 months 22.

Unfortunately, a large number of patients still experience disease recurrence even after complete surgical resection. Adjuvant mitotane treatment is associated with increased disease-free survival but not OS 30. There are discussions regarding how long mitotane should be used in this setting, but most specialists agree that 2 years should be the minimum period. However, mitotane is toxic, and many patients do not tolerate treatment for long periods. Thus, adjuvant mitotane should be considered mainly for high-risk patients (stage III disease or tumors >8-10 cm in size, those with high mitotic rates or a proliferative index Ki67 >10%, and those with microscopic evidence of vascular or capsular invasion). The benefit of mitotane in the low/intermediate-risk group of patients is currently being evaluated in the multicentric, prospective, phase III ADIUVO trial 2. Adjuvant radiation therapy of the adrenal bed is associated with a reduction in local disease recurrence only, with no benefits in OS 31. Few retrospective studies have shown that radiation therapy may improve local control after surgical resection in patients with localized ACCs 31-34. Therefore, the indications for adjuvant radiation therapy are still being debated.

Mitotane is the only available adrenolytic drug for ACC treatment. In addition to its use in the adjuvant setting, mitotane is associated with a 30% objective response rate among patients with metastatic disease in retrospective studies. Mitotane inhibits steroidogenesis and controls hormone excess syndromes. Treatment should be initiated at a dose of 1.5 g/d, with a progressive increase up to 5-6 g/d. The goal during treatment is to achieve plasma mitotane levels between 14 and 20 mcg/mL 35. Plasma mitotane concentrations greater than 20 mcg/mL are associated with significant toxicity, including neurological effects (lethargy, somnolence, ataxia, and polyneuropathy, among others). Plasma concentration monitoring should be performed every 4 to 6 weeks during the initiation of treatment. Most patients require at least 2 to 3 months of treatment to achieve therapeutic levels 35.

The most common side effects of mitotane are gastrointestinal and are characterized by anorexia, nausea, vomiting, diarrhea and abdominal discomfort. Mitotane also increases the concentrations of cortisol-binding globulin hormone (CBG), steroid-binding globulin (SHBG), and thyroxine-binding globulin (TBG). Furthermore, mitotane may impair pituitary gland function by reducing TSH secretion with consequent hypothyroidism and gonadal function causing hypergonadotropic hypogonadism 23. Cholesterol and triglyceride levels should be monitored regularly due to increases in LDL-cholesterol, HDL-cholesterol and triglycerides 35. In the presence of persistently elevated lipid concentrations, statin use is indicated. Statins that are not metabolized by CYP3A4 are preferable, such as rosuvastatin and pravastatin.

Notably, mitotane induces adrenal insufficiency due to the inhibition of steroidogenesis enzymes. As mitotane also increases peripheral cortisol metabolism and increases CBG concentrations, patients on glucocorticoid replacement therapy need higher replacement doses. The indications for mineralocorticoid replacement depend on potassium and renin levels 35.

Chemotherapy has been used but with limited results. Recently, the first randomized phase III trial with metastatic ACC patients compared to two promising protocols (etoposide, doxorubicin, cisplatin and mitotane [EDPM] *vs.* streptozotocin and mitotane) 36. EDPM proved to be superior in terms of the objective response rate (20.5% *vs.* 7.9%) and progression-free survival (5 m *vs.* 2.1 m) but was not superior in terms of OS. This study established EDPM as the most evidence-based first-line therapy but also highlighted the necessity for better therapies to be developed for ACC patients 36. A phase I study of imatinib, dacarbazine, and capecitabine included 6 patients with adrenal cancer 37. Among ACC patients, one of the 6 patients presented a partial response with a progression-free survival of 8.8 months 37.

Several phase II studies have investigated molecular target therapies in patients with advanced ACC: inhibitors of epidermal growth factor receptor (EGFR; erlotinib and gefitinib), mammalian target of rapamycin (mTOR; everolimus), platelet-derived growth factor receptor (PDGFR) and c-KIT (imatinib), and vascular endothelial growth factor (VEGF; bevacizumab) and antibodies against insulin-like growth factor 1 receptor (IGF-1R; figitumumab and cixutumumab) did not demonstrate any effectiveness 38. Linsitinib, an oral IGF-1R inhibitor, was evaluated in a randomized placebo-controlled phase III study including 139 patients with advanced ACC 39. Unfortunately, linsitinib did not improve progression-free survival or OS when compared to placebo.

Surgical debulking can be considered in patients with nonresectable tumor masses, limited sites of metastasis or clinical findings of extreme hormonal excess that prove to be refractory to clinical treatment. Although not completely proven, surgical debulking might offer survival advantages for patients with metastatic disease 29.

### Malignant pheochromocytoma and paraganglioma

Pheochromocytomas and paragangliomas (PPGLs) are neuroendocrine tumors derived from chromaffin cells of the adrenal medulla (called pheochromocytomas) or from the sympathetic or parasympathetic autonomic ganglia (paragangliomas). Approximately 80 to 85% of chromaffin-cell tumors are pheochromocytomas, whereas 15 to 20% are paragangliomas. In this review, pheochromocytomas and paragangliomas are referred to as PPGLs. These tumors account for up to 0.6% of cases of adult hypertension and 1% of cases of pediatric hypertension 6. More than 30% of patients with PPGLs have a hereditary predisposition, and up to 50% of patients with metastatic disease have hereditary germline mutations. To date, at least 14 tumor susceptibility genes have been described 6,40,41. These susceptibility genes can be divided into two clusters: cluster 1 tumors include those with mutations of genes encoding the von Hippel-Lindau (VHL) suppressor, the four subunits of the succinate dehydrogenase complex (SDHA, SDHB, SDHC, and SDHD), and less commonly, the enzyme responsible for flavination of the SDHA subunit (SDHAF2), fumarate hydratase, malate dehydrogenase 2, and prolyl hydroxylases 1 and 2. These genetic alterations result in stabilization of hypoxia-inducible factors and activation of the hypoxia signaling pathways. Cluster 2 includes tumors with mutations of the neurofibromatosis type 1 (NF1) tumor suppressor gene, the rearranged during transfection (RET) proto-oncogene, genes encoding transmembrane protein 127 (TMEM127) and MYC-associated factor X (MAX) 6,40,41 (Table 3). We recommend genetic screening for PPGL patients in the following situations: those with bilateral pheochromocytomas, all patients with paragangliomas, those with malignant PPGLs and patients younger than 45 years with isolated pheochromocytomas 6,42.

### Diagnosis of malignant PPGLs

The early diagnosis of PPGLs is crucial for several reasons. Most PPGLs hypersecrete catecholamines and are associated with increased cardiovascular morbidity and mortality. Additionally, PPGLs may cause mass-effect symptoms and have malignant potential. Additionally, familial counseling may result in earlier diagnosis and treatment of asymptomatic carriers. Malignancy is defined by metastases in nonchromaffin tissue. Malignancy in PPGLs affects between 10 and 17% of patients, but the prevalence of such malignancy depends mostly on the germline mutation underlying PPGL pathogenesis 6. Approximately 35% of patients present with synchronous metastases, whereas 65% of patients develop metachronous metastases at a median of 5.5 years after diagnosis 43. Patients who are young and those with larger tumors (>6 cm), positive genetic testing (especially *SDHB*) or paragangliomas have an increased risk of metastasis and require long-term follow-up. Patients with negative genetic testing and pheochromocytomas <4 cm in size have a lower risk of malignancy 44. In a recent study, 76% of patients with malignant PPGLs had germline mutations in susceptibility genes for PPGL (*SDHB*, 44%; *SDHD*, 8%; *VHL*, 12%; *NF1*, 12%; and *RET*, 0%) 44. *TMEM127* and *MAX* mutations have also been described in patients with malignant PPGLs 45.

Biochemical investigation of PPGL patients should include measurement of plasma free metanephrine or 24-h urinary fractionated metanephrine concentrations using liquid chromatography with mass spectrometry. Total 24-h urinary catecholamine concentrations should also be measured to avoid missing isolated dopamine-producing PPGLs. To measure plasma metanephrines, blood should be drawn with the patient in the supine position. Topographical investigation for PPGLs should be performed only after the biochemical diagnosis of PPGLs. Imaging studies should be initiated with abdominal CT or MRI 6.

The sensitivity of ^123^ I-metaiodobenzylguanidine (MIBG) scintigraphy is approximately 80% for pheochromocytomas and 50% for paragangliomas 46,47. ^123^ I-MIBG is recommended in patients with an increased risk for metastatic disease due to the large size of the primary tumor, positive genetic testing or extra-adrenal, multifocal or recurrent disease 6. For high-risk patients, we also recommend chest CT to identify lung metastasis. Recent studies have demonstrated that ^123^I-MIBG SPECT is similar to PET-CT using ^18^F-FDG to detect PPGLs 48. ^123^I-MIBG is inferior to ^18^F-FDG-PET or somatostatin receptor imaging for paragangliomas or metastatic disease associated with *SDHx*-related tumors 49.

### Treatment

All patients with a hormonally functional PPGLs should be treated with α-adrenergic receptor blockers to prevent perioperative cardiovascular complications. Preoperative medical treatment for PPGL patients should be initiated at least 14 days before surgery to allow adequate time to normalize blood pressure and heart rate. Since PPGL patients have severe vasoconstriction with volume depletion, treatment should include a high-sodium diet and fluid intake to prevent severe hypotension after tumor removal 6. Selective α_1_-receptor blockers (extended-release prazosin or doxazosin) to control blood pressure and adrenergic symptoms are recommended. The dose should be started at 1 mg twice daily and titrated to 10-14 mg/d. Preoperative β-adrenergic receptor blockers are indicated to control tachycardia after the administration of α-adrenergic receptor blockers. The use of β-adrenergic receptor blockers should not be initiated before α-adrenoceptor blockers because the unopposed stimulation of α-adrenergic receptors can induce adrenergic crises. If the patient has been α-blocked and remains hypertensive, calcium blocker channels should be added. The treatment goal is a blood pressure of less than 130/80 mmHg while seated and greater than 90 mmHg systolic while standing. Furthermore, α-receptor blockers should be discontinued 12h before surgery to prevent refractory hypotension after tumor removal. We recommend α-blockade even in normotensive patients if the PPGL is biochemically active.

The treatment of choice for localized PPGLs is laparoscopic resection. Open resection is indicated for tumors >6 cm in size or invasive PPGLs to ensure complete tumor resection, prevent tumor rupture, and avoid local recurrence. Patients with pheochromocytomas larger than 6 cm or paragangliomas of any size and/or those who are carriers of *SDHB* mutations need imaging studies to localize metastatic disease 50. For high-risk patients, we recommend abdominal and pelvic MRI and chest CT every 4-6 months during the first 2-3 years and then annually. Ideally, a whole-body scan, such as an ^18^F-FDG-PET scan, should be performed for patients with *SDHB*-positive tumors every 1 or 2 years in addition to routine imaging evaluations.

In a recent retrospective study from the Mayo Clinic, the median overall and disease-specific survivals for malignant PPGLs were 25 and 34 years, respectively. These findings indicate a slow disease course in a subgroup of patients. Shorter survival was correlated with male sex, older age at diagnosis, synchronous metastases, larger primary tumor size, elevated dopamine levels and not undergoing primary tumor resection 43. Patients with metastatic disease and biochemically active tumors should remain α-blocked during the follow-up period. Approximately 70% of patients with malignant PPGLs develop bone metastases (mainly lytic). In all patients with bone metastases, the use of antiresorptive drugs (zoledronic acid every 6 months) may be considered to avoid skeletal-related events 51. Locoregional guided therapy, such as surgery and interventional radiology, may be indicated to control pain and prevent fractures and spinal cord compression.

Since the long-term survival is more than 10 years in a subgroup of patients, and systemic therapies have substantial toxicity, the initiation of systemic therapy for malignant PPGL patients is justified mainly by tumor progression as defined by RECIST (www.recist.com) (Table 4). The most studied targeted therapy for malignant PPGLs is ^131-^I-MIBG therapy. Because the structure of MIBG is similar to that of noradrenaline, this isotope can be taken up by tumor cells and cause radiation-induced cell death. Approximately 50% of patients with metastatic lesions have a positive MIBG uptake. Therefore, ^131^I-MIBG therapy is the first-line therapy for this subgroup of patients. MIBG therapy is associated with response rates ranging from 22 to 48% 50. A single phase II trial included 50 patients with malignant PPGLs who were treated with 131I-MIBG doses ranging from 492 to 1,160 mCi 52. The objective response rate was 22%. Disease progression was found in 35% of patients after a year of follow-up. The estimated 5-year OS rate was 64%. Toxicities included grade 3 to 4 neutropenia (87%) and thrombocytopenia (83%). Two small studies of ^90^Y-DOTATOC and ^177^Lu-DOTATOC showed tumor response rates of 8% and 17%, respectively 53,54.

A retrospective study from the MD Anderson Cancer Center reported an objective response rate of 25% using cyclophosphamide and dacarbazine and the optional use of vincristine or doxorubicin in 52 patients with malignant PPGLs 55. In addition, a Japanese study reported a partial or minimal tumor response in 8 (47%) out of the 17 patients with malignant PPGLs treated with cyclophosphamide, vincristine and dacarbazine 56. Temozolomide led to a response rate of 33% in patients with malignant PPGLs 57. Regarding molecular target therapy, patients with malignant PPGLs treated with everolimus had no response 58,59. Sunitinib promoted an objective response in 3 (18%) out of 17 malignant PPGL patients, although 43% of the patients had improvement in blood pressure control. In this study, most of the patients who exhibited a positive response to sunitinib therapy had *SDHB* germline mutations 60.

### Final remarks

Surgery is the only therapy that offers a cure for both ACCs and PPGLs. High-risk ACC and PPGL patients should be carefully monitored for recurrence. Among PPGL patients, *SDHB* and *SDHD* mutation carriers have a significant risk of recurrence. Since current treatments for both metastatic ACCs and PPGLs are not associated with a good objective response rate, trials of adjuvant therapies for high-risk patients might be the most promising strategies to decrease recurrence and improve OS. In addition, advanced metastatic ACCs and PPGLs should be preferably addressed in prospective clinical trials testing new molecular target agents.

## AUTHOR CONTRIBUTIONS

Almeida MQ and Fragoso MC wrote the manuscript. Latronico AC, Mendonça BB and Bezerra-Neto JE reviewed the manuscript and provided critical feedback about this manuscript.

## Figures and Tables

**Table 1:** Inherited disorders associated with ACC [Adapted from Patsy et al. (14).

Syndrome*	Gene mutation	General population prevalence	Prevalence in ACC patients	Other characteristics
LFS	*TP53*	1:20.000	3-7% (adults) 50-80% (children)	Sarcomas, choroid plexus tumors, cerebral tumors, breast cancer, leukemia, lymphoma.
MEN1	*MEN1*	1:30 000	1-2% (adults)	Neuroendocrine tumors, pituitary tumors, parathyroid hyperplasia, adrenal adenomas, angiofibromas.
Lynch	*MSH2, MSH6, MLH1, PMS2*	1:440	3% (adults)	Colorectal cancer, endometrial cancer, ovarian cancer, pancreatic cancer, brain tumors.
BWS	*IGF2, CDKN1C*	1:13.000	Very rare (children)	Wilms’ tumors, hepatoblastomas, adrenal adenomas, macrosomia, macroglossia, omphaloceles.
FAP	*APC*	1:30.000	<1%	Colonic polyposis, colorectal cancer, desmoid tumors, thyroid cancer, adrenal adenomas, congenital retinal hyperplasia, epidermoid cysts.
NF1	*NF1*	1:3.000	<1%	Pheochromocytomas, café-au-lait spots, neurofibromas, optic gliomas, Lisch nodules, skeletal abnormalities.

*LFS: Li-Fraumeni syndrome, MEN1: Multiple endocrine neoplasia type 1, BWS: Beckwith-Wiedemann syndrome, FAP: Familial adenomatous polyposis, NF1: Neurofibromatosis type 1.

**Table 2:** Imaging characteristics of the most prevalent tumoral lesions in the adrenal gland (23).

**Adrenal Adenoma** Frequently <4 cm, homogeneous and well-defined in shape CT: density <10 HU* without contrast; 40 to 50% washout or density <35 HU 10-15 min after contrast injection MRI: homogenously isointense or hypointense on T2-weighted sequences.
**Adrenal Carcinoma** Frequently >6 cm, heterogeneous and irregularly shaped. CT: density >10 HU without contrast; less than 40 to 50% washout or density >35 HU 10-15 min after contrast injection MRI: hyperintense on T2-weighted sequences; <30% loss of signal intensity on out-of-phase images.
**Metastasis** Heterogeneous lesions, frequently affecting both glands, with contrast enhancement. CT and MRI: similar findings to adrenal carcinomas
**Pheochromocytoma** Heterogeneous and dense lesions with intense contrast enhancement. MRI: hyperintense on T2-weighted sequences; <30% loss of signal intensity on out-of-phase images.
**Myelolipoma** CT: Import lipid content with very low density MRI: similar behavior as lipid content in the subcutaneous tissues and retroperitoneum on phase-sequences.

*Hounsfield unit.

**Table 3:** Frequency of germline mutations in 519 adults with PPGLs and risk of malignancy (42).

Genes	Frequency (%)	Risk of malignancy in mutation carriers (%)
*Cluster 1*		
*VHL*	10	6
*SDHB*	17	69
*SDHD*	11	31
*SDHC*	1	17
*Cluster 2*		
*RET*	9	4
*NF1*	4	5

**Table 4:** Objective response rate after systemic therapy for malignant PPGLs.

Treatment	Study	n	Objective response rate (%)	Reference
^131^I-MIBG	Phase II	50	22	(52)
^90^Y-DOTATOC	Phase II	25	8	(53)
^177^Lu-DOTATOC	Phase II	12	17	(54)
Cyclophosphamide and dacarbazine*	Retrospective	52	25	(55)
Cyclophosphamide, vincristine and dacarbazine	Retrospective	17	47	(56)
Temozolomide	Retrospective	15	33	(57)
Everolimus	Phase II	7	0	(59)
Sunitinib	Retrospective	17	18	(60)

*Optional use of vincristine or doxorubicin.
